# Synchronous shedding of multiple bat paramyxoviruses coincides with peak periods of Hendra virus spillover

**DOI:** 10.1080/22221751.2019.1661217

**Published:** 2019-09-07

**Authors:** Alison J. Peel, Konstans Wells, John Giles, Victoria Boyd, Amy Burroughs, Daniel Edson, Gary Crameri, Michelle L. Baker, Hume Field, Lin-Fa Wang, Hamish McCallum, Raina K. Plowright, Nicholas Clark

**Affiliations:** a Environmental Futures Research Institute, Griffith University, Nathan, Queensland, Australia; b Department of Biosciences, Swansea University, Swansea, Wales, UK; c Department of Epidemiology, Johns Hopkins University Bloomberg School of Public Health, Baltimore, MD, USA; d CSIRO, Health and Biosecurity Business Unit, Australian Animal Health Laboratory, Geelong, Vic, Australia; e Department of Agriculture, Animal Health Policy Branch, Canberra, ACT, Australia; f EcoHealth Alliance, New York, NY, USA; g School of Veterinary Science, The University of Queensland, Gatton, Queensland, Australia; h Programme in Emerging Infectious Diseases, Duke-National University of Singapore Medical School, Singapore; i Department of Microbiology and Immunology, Montana State University, Bozeman, Montana, USA; j UQ Spatial Epidemiology Laboratory, School of Veterinary Science, the University of Queensland, Gatton, Queensland, Australia

**Keywords:** Pteropus, emerging infectious diseases, multi-viral, viral communities, zoonoses, disease ecology, co-occurrence analyses, Markov Random Fields

## Abstract

Within host-parasite communities, viral co-circulation and co-infections of hosts are the norm, yet studies of significant emerging zoonoses tend to focus on a single parasite species within the host. Using a multiplexed paramyxovirus bead-based PCR on urine samples from Australian flying foxes, we show that multi-viral shedding from flying fox populations is common. We detected up to nine bat paramyxoviruses shed synchronously. Multi-viral shedding infrequently coalesced into an extreme, brief and spatially restricted shedding pulse, coinciding with peak spillover of Hendra virus, an emerging fatal zoonotic pathogen of high interest. Such extreme pulses of multi-viral shedding could easily be missed during routine surveillance yet have potentially serious consequences for spillover of novel pathogens to humans and domestic animal hosts. We also detected co-occurrence patterns suggestive of the presence of interactions among viruses, such as facilitation and cross-immunity. We propose that multiple viruses may be interacting, influencing the shedding and spillover of zoonotic pathogens. Understanding these interactions in the context of broader scale drivers, such as habitat loss, may help predict shedding pulses of Hendra virus and other fatal zoonoses.

## Introduction

Natural systems comprise complex host-parasite communities, with individuals normally co-infected with multiple macro and microparasite species [1]. Co-infecting pathogens can influence exposure [2], infectiousness [3–5], susceptibility to, and mortality from [6,7], other pathogens [8,9]. Yet, despite the ubiquity of co-infections, research remains biased towards single host–single parasite frameworks. Studies that recognize the multivariate nature of parasite communities often focus on interactions across broad taxonomic groups (e.g. helminth-viral or bacterial-viral co-infection [10]) or on infection with multiple strains of a single viral species [11]. With a few exceptions (e.g. HIV [12], some respiratory viruses [13] and avian influenza [14]), co-infection studies of interactions among and within viral families are rare. While this has been, in part, due to technical challenges in the past, the advance of highly multiplexed approaches and discovery-driven tools is opening up opportunities to enter into this important field of research.

Bats are hosts to some of the most significant emerging zoonoses, including Ebola, Marburg, SARS, Nipah, and Hendra viruses [15], yet the incidence of viral co-infections in bats is poorly understood. Cross-species transmission (spillover) of viruses from bats to other mammals occurs worldwide [16]. If viral co-infections influence or reflect variation in host exposure, infectiousness, or susceptibility as in other host-parasite systems, moving beyond studies of single-pathogen-single host dynamics to viral community dynamics could progress our understanding of transmission and spillover of bat pathogens.

Hendra virus (HeV; Family *Paramyxoviridae*, Subfamily: *Orthoparamyxovirinae*, Genus *Henipavirus*) in Australian flying foxes is a well-studied model system for understanding bat virus transmission and spillover globally [17]. While it appears not to result in any ill-health for bat hosts, HeV infection is fatal for horses and humans[18]. The virus is known to circulate widely in Australian flying foxes [19], with black flying foxes (*Pteropus alecto*) and spectacled flying foxes (*P. conspicillatus*) considered the primary reservoir hosts [20,21]. Viral shedding is primarily via urine [20], and fatal spillover to horses tends to be associated with seasonal “pulses” of viral shedding from bat populations [19]. Environmental drivers of transmission are also expected to play a role in HeV shedding and spillover. For example, viral survival is prolonged in winter periods [22], and HeV prevalence in flying foxes and spillover to horses has been associated with cold, dry seasonal conditions [23,24] and nectar scarcity [25] in the subtropics. Despite these recent advances in understanding, interactions among landscape-scale and roost-scale drivers of HeV transmission remain unclear [26] and spillover from bats to horses remains difficult to predict.

Research on bat viruses in Australia has largely focused on HeV, yet a diverse community of other viruses has been detected [27–32] (Table 1), with little knowledge about their transmission dynamics, host range or zoonotic potential. The likelihood of a bat being infected with each of these viruses is likely influenced not only by host ecology and specific immune responses to each viral species, but also by potential facilitative or antagonistic interactions between pairs of viruses within the broader viral community [8]. Examining the spatiotemporal patterns of viral co-occurrence within populations (*co-circulation*) and within individual hosts (*co-infection*) is likely to provide much-needed further insights into underlying processes driving viral community dynamics.

**Table 1.** Details of known flying fox paramyxoviruses included in this study.

Genus	Species	Abbreviation	Known to occur in Australia	Reference
*Henipavirus*	Cedar	CedV	Yes	[28]
*Henipavirus*	Hendra	HeV	Yes	[18]
*Henipavirus*	Nipah	NiV	No	[33]
*Pararubulavirus*	Menangle	MenV	Yes	[32]
*Pararubulavirus*	Teviot	TevPV	Yes	[27]
*Pararubulavirus*	Tioman	TiV	No	[34]
*Unclassified*	Geelong	GeePV	Yes	Unpublished. High sequence similarity to Alston virus [31]
*Unclassified*	Grove	GroPV	Yes	[27]
*Unclassified*	Hervey	HerPV	Yes	[27]
*Unclassified*	Yarra Bend	YBPV	Yes	Unpublished
*Unclassified*	Yeppoon	YepPV	Yes	[27]

Theoretical predictions of viral community dynamics at the population level have rarely been tested with empirical data (but see, [35]), and certainly not among bat viral communities. Here, we examine synchronies in virus shedding from flying fox populations sampled over time and space and compare rates of viral co-detection at the sample and roost-level. Specifically, we use Markov Random Fields to (1) detect patterns of paramyxovirus co-occurrence in Australian flying foxes and (2) test whether these patterns can be explained by positive or negative virus-virus interactions or regional environmental conditions. Ultimately, we aim to assess whether the better understanding of viral community interactions and dynamics can elucidate drivers of viral dynamics and spillover.

## Materials and methods

### Sample collection

**Figure 1.**
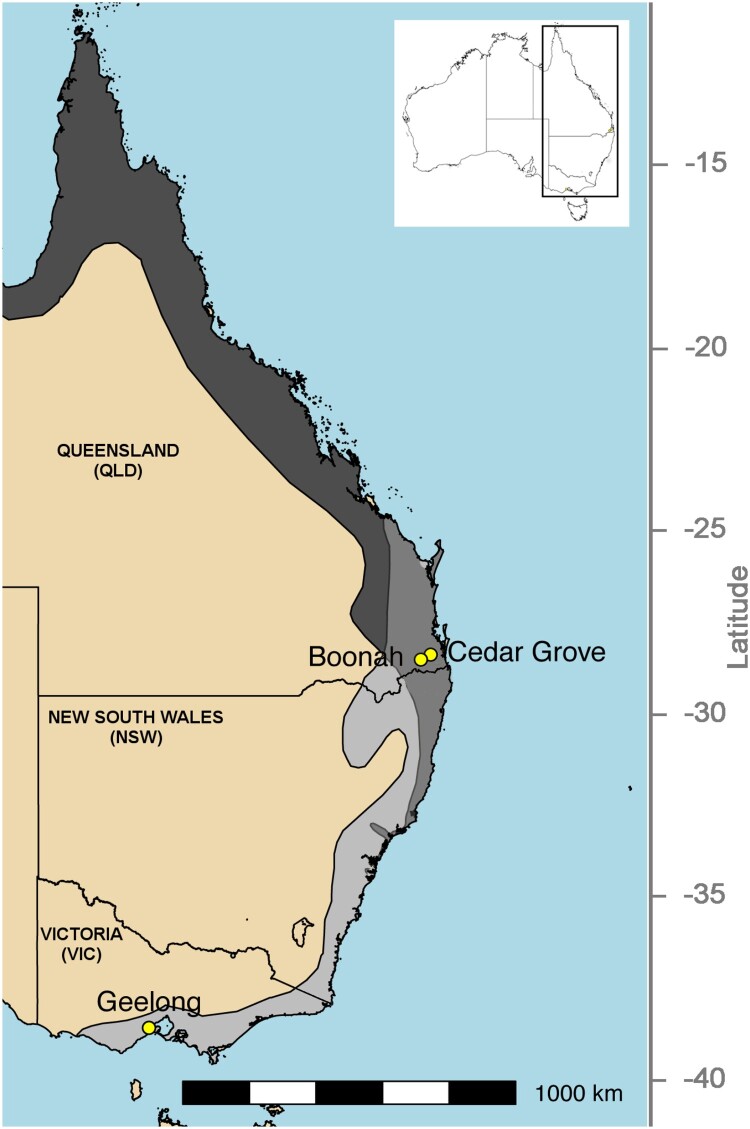
Map showing sampling sites in Queensland and Victoria, as well as species distributions for *Pteropus alecto* (dark grey) and *P. poliocephalus* (light grey). The distributions of these species overlap in southeast Queensland and northeast New South Wales (medium grey). Inset shows the location of the study area within Australia.

Between November 2010 and November 2012, urine samples were collected at flying fox roost sites in Queensland (QLD) and Victoria (VIC) as previously described [19,36] (Figure 1, Table S1). At the time of sampling, the QLD roost sites (Boonah and Cedar Grove) generally contained mixed roosts of black flying foxes (*P. alecto*) and grey-headed flying foxes (*P. poliocephalus*), with occasional small numbers (<0.5%) of little red flying foxes (*P. scapulatus*), whereas, in VIC (Geelong), one single species roost of grey-headed flying foxes was sampled (Table S1). Animal ethics and related approvals are detailed in the original studies [19,36]. Briefly, each sampling session comprised placing either ten (QLD) or four (VIC) 3.6 m x 2.6 m plastic sheets underneath occupied trees within each roost, typically pre-dusk. The following morning at dawn, up to five urine samples were collected from discrete areas of each sheet to minimize the likelihood that an individual bat contributed to multiple pools. Up to 30 samples were collected during each sampling session. It is likely that multiple flying foxes contributed to each sample.

### Sample analyses

To provide information on the possible presence of multiple viral species in each sampling session, viral RNA in the under-roost urine samples was amplified and tested using multiplex fluid bead assay using primers specific to each of 11 known bat paramyxoviruses, as previously described (See Supplementary methods and Table S2; [37]). This assay was developed based on the known sequences of the viruses included in the panel and primers and probes were tested to exclude the possibility of any cross PCR amplification with any other viral member of the whole panel. Based on prior analyses [37], we defined a urine sample positive for the target viral RNA when the Median Fluorescence Intensity (MFI) was greater than 400 fluorescence units.

### Estimating viral co-occurrence probabilities

Co-occurrence analyses (such as Markov Random Fields [38,39]) test whether a species’ occurrence covaries with occurrences of other species, which makes them particularly well-suited to quantifying the drivers of community composition [40–42]. Pairwise co-occurrence probabilities may be important precursors for, or indicators of, biotic interactions such as cross-immunity-induced competition and immunosuppression-related facilitation [43–45]. Applied to viruses, co-occurrence analyses can also highlight species that respond similarly to underpinning environmental conditions and demographic dynamics, but independent of interactions among virus species [41]. Markov Random Fields represent pairwise conditional dependencies (here referred to as “interactions”) among co-occurring species in an undirected graphical network. A key advantage of Markov Random Fields is that they represent undirected relationships, allowing inference on how interactions between two species are conditional on their respective relationships with all other species. This represents a step forward compared to competing methods, many of which require comparisons to unrealistic null models and do not adequately account for indirect relationships [46,47].

We estimated Markov Random Field networks using viral presence/absence data at two levels of data aggregation. Firstly, we considered the *sample level*, with each pooled sample comprising 10–20 discrete urine droplets collected from a discrete area on a sheet placed underneath roosting bats (*N* = 1131). While it is probable that multiple individuals contributed to each urine sample collected, for this under-roost urine collection approach, this aggregation level best approximates urine samples collected at the individual level. Secondly, we considered the *session level*, representing an aggregation of presence/absence data from all samples tested within each sampling session (*N* = 98). This session-level aggregation approximates the observed presence-absence of viruses in populations at a given time. We compared the direction of Markov Random Field coefficients for all pairs of virus species across hierarchical levels of data aggregation and generated inferences about possible co-infection/co-circulation of bat-borne viruses. Differences in viral pair associations between sample and session levels may reflect signatures of co-infection (viral co-occurrence within a host) versus co-circulation (viral co-occurrence within populations) and may be useful for generating insights into viral-species or host interactions. We used functions in the *rosalia* R package to estimate viral co-occurrences, as this package offers Maximum Likelihood utilities to solve small Markov Random Field networks (up to ∼ 20 species) and can approximate the network’s gradient for communities with larger numbers of species [48].

### Exploring environmental affinities of viruses using logistic regressions

We supplemented Markov Random Field analyses by fitting generalized linear models (GLM; specifying a binomial error and logit link function) using viral presence/absence vectors from the session level (*N* = 98) to quantify associations between virus occurrence probabilities and biotic and abiotic covariates. Three abiotic covariates were included based on previous research showing that time-lagged variables describing vegetation and climate within the foraging radius of roosting sites (∼20 km) are drivers of flying fox ecology [25,49] and virus survival in the environment [22]. The abiotic covariates include mean Normalized Difference Vegetation Index (NDVI) within 20 km over the preceding 3 months, mean precipitation in the preceding month, and mean water vapour pressure on the day of sampling. We included both presence/absence and counts (a coarse proxy of relative abundance) of roosting bat species as biotic covariates because a previous study found relative bat abundance to be associated with HeV shedding in bat populations [23]. We also accounted for temporal variation across the three sampling years by including year as a categorical covariate. Starting with full models that included all the aforementioned covariates, we used backward stepwise variable selection to reduce the parameter space and eliminate covariates that did not improve model fit (e.g. inclusion of the covariates did not significantly improve fit based on an analysis of deviance). GLM fits and stepwise model selection were carried out using functions in the base R software.

## Results

### Multiple paramyxoviruses are co-circulating in Australian flying foxes across broad spatiotemporal scales

In total, we recorded nine viral species (out of 11 targeted species) from 1,131 pooled under-roost urine samples, rendering 12,441 unique observations of virus presence or absence over 98 sampling sessions based on MFI values. RNA from all nine viruses was detected from Boonah (*N* = 320 samples) (Figure S1), with 8/9 detected within weeks of a HeV spillover (fatal transmission event from a flying fox to a horse) associated with this roost. With a smaller sample size (*N* = 129 samples), only three viruses were detected at Cedar Grove (MenV, GroPV and TevPV). Despite extensive sampling (*N* = 682 samples over a 20-month period), only five viruses were detected at the southernmost site in Geelong, Victoria (GeePV, YBPV, TevPV, CedV, YepPV; Figure S1). Tioman virus and NiV were not detected. Hendra virus detection sensitivity differed in the multiplexed fluid-based assay compared to a quantitative RT-PCR (RT-qPCR) HeV assay (reported in [19]). Of 63 samples that were positive in the RT-qPCR assay, only 14 were positive in the multiplexed fluid-based assay. However, there was a highly significant linear relationship between natural log transformed MFI (lnMFI) and Ct values, indicating that viruses present in low viral loads were likely undetected by the multiplexed fluid-based assay (Figure S2). We therefore acknowledge that detections here are underestimates of true shedding.

### Viral detections were highly spatially and temporally clustered

**Figure 2.**
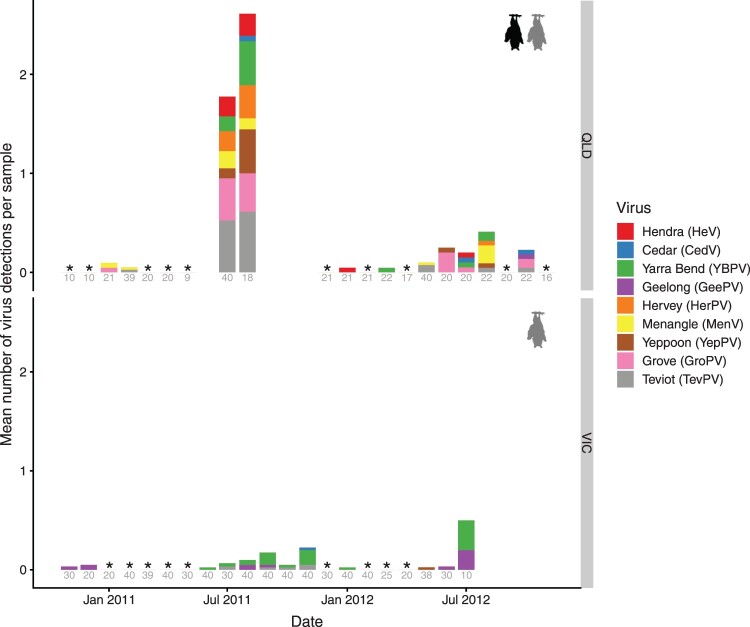
Average number of viral detections per urine sample for nine paramyxoviruses (colours) from two flying fox roosts in Queensland (QLD) and one in Victoria (VIC) in different months. Some months comprise multiple sampling events (see Table S1). In QLD, dates prior to June 2011 represent sampling from Cedar Grove, and after this date, sampling continued from the nearby, newly formed Boonah roost. * = sampled but no detections, blanks = not sampled. Grey numbers represent sample sizes. QLD sites contained a mix of black flying foxes (*P. alecto*), grey-headed flying foxes (*P. poliocephalus*) and occasional small numbers (<0.5%) of little red flying foxes (*P. scapulatus*), whereas VIC contained only *P. poliocephalus*.

Each virus generally circulated at low or undetectable levels (e.g. HeV was only detected in 5/25 months and HerPV in 3/25 months surveyed; Figure 2) and overall viral prevalence was <5% for all viral species (Figure S3). Viral detections occurred most commonly within “pulses” of multi-species viral shedding over multiple consecutive months as well as intermittently in isolation from other viruses (Figures 2, S4). A particularly high peak in virus shedding was observed in Boonah in winter 2011 (Figures 2, S4): viral RNA was detected from up to 6 unique viruses per sample (mean of 1.8). In QLD, detections peaked between June and October (centred around July-August, though this was overwhelmingly driven by winter 2011), whereas the peak was later (July-November) and less pronounced in VIC (Figure S5). Some viruses (TevPV, YBPV) were detected across all seasons, whereas HerPV was only detected in winter.

### Viral co-occurrence patterns suggest potential facilitation and cross-neutralisation

**Figure 3.**
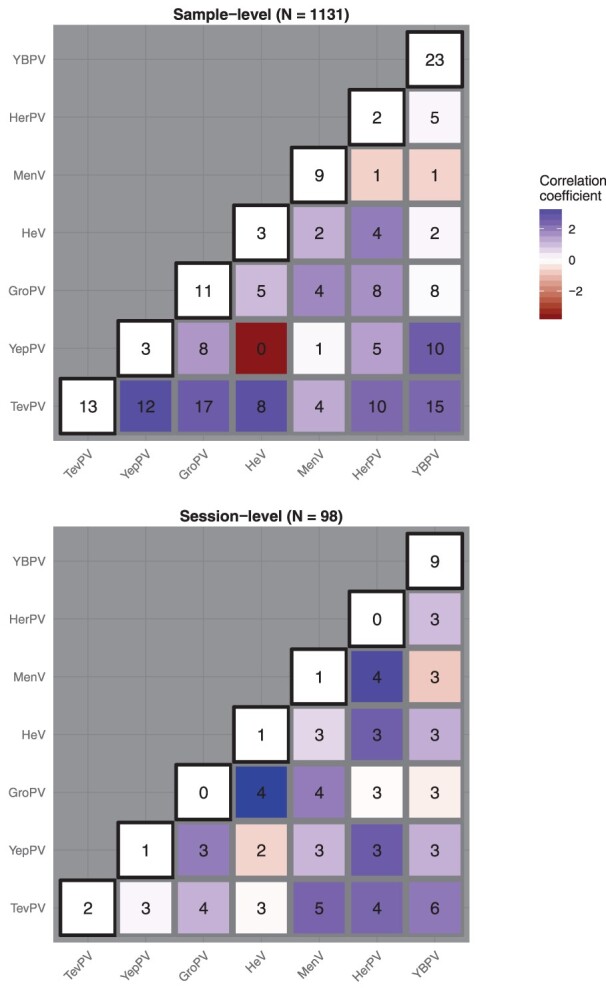
Viral occurrence and interaction coefficients at the sample (top) and session (bottom) level. Colours represent predicted interactions from a Markov Random Fields model fit without any additional covariates. Positive interactions are in blue; negative interactions in red. Values on the diagonal (with black borders) represent the number of single detections observed for each of seven paramyxoviruses (virus abbreviations as per Table 1). Off-diagonal values represent the total number of co-detections observed for each virus pair. Note that values do not add to totals in headings (the total number of samples = 1,131, or total number of sessions = 98), as some samples/sessions exhibited co-detections of RNA for three or more viruses. Note, correlations represent regression coefficients for a virus’ log-odds and therefore are not restricted to [0, 1].

Two viruses (CedV and GeePV) were included in co-occurrence analyses as predictors rather than as response variables, as these viruses were rare (*N* = 4 and *N* = 9 total detections, respectively), and as such would be difficult to estimate accurately. For the remaining seven viruses, we detected a number of well-supported, mainly positive, conditional associations among viruses at the sample and session levels (Figure 3). At the sample level, TevPV showed the strongest pairwise and highest mean interaction coefficients, suggesting that detection of TevPV within a sample consistently increased the likelihood of co-detection of other viruses (Figure 3 and Figure S6). HeV and YepPV were strongly negatively associated with each other at the sample level, with no co-detections in the same samples and only two co-detections within the same sessions (Figure 3).

**Figure 4.**
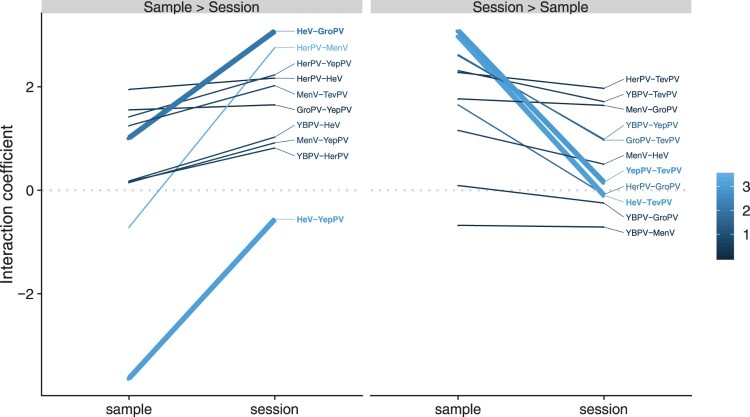
Difference in sample- and session-level viral interaction coefficients from a Maximum Likelihood Markov Random Fields model. Virus pairs were divided into those where the interaction coefficient was higher at the session level than sample level (inclining slope, left plot) and vice versa. The grey dotted line at zero divides the plot into positive pairwise interactions (top) and negative pairwise interactions (bottom). Steep downward slopes (sample interaction >> session interaction) are consistent with co-infection or facilitation. Steep upward slopes are consistent with co-circulation, with the exception of HeV-YepPV, which showed strong negative interactions within samples (indicative of competition). In the colour scale, darker colours represent a greater absolute difference between sheet and sample level coefficients. Virus pairs with thicker lines are discussed in more detail in the text. Virus abbreviations as per Table 1.

Some virus pairs were positively associated at all hierarchical levels of analysis, but the strength of those associations varied across levels. For example, HeV and GroPV were highly likely to be detected in the same sessions but were less frequently co-detected in the same samples (Figure 4, left panel). The strong positive interaction at the highly aggregated session level alongside weaker positive interaction at the sample level (which more closely resembles individual infection status) may be indicative of co-circulation of the two viruses associated with shared drivers of environmental or temporal affinities. In this case, synchronous drivers of transmission at the population level would drive increased likelihood of co-detection within samples by chance, producing the weaker sample-level interactions. Conversely, TevPV showed no signal of association with either HeV or YepPV at the session level, but on those occasions when they did co-occur, TevPV was highly likely to be detected within the same samples as either HeV or YepPV (Figure 4, right panel, Figure S7). This could reflect co-infection within individual bats or co-circulation among individuals roosting in immediate proximity, so that they contribute urine to the same pool. Interestingly, there was also no association between HeV and YepPV at the session level, but a strong negative association between them at the sample level (Figure 4, left panel). This potentially suggests that individuals infected with TevPV are likely to be co-infected with either HeV or YepPV, but not both. These processes could be acting at an individual level, reflecting within-host viral competition mediated by cross-reactivity, or at a species level, reflecting host-specificity.

### Viruses shared similar environmental affinities

Although virus detections were fairly sparse, the clustered nature of detections provided additional information about whether shared environmental associations can explain co-occurrence at the session level. The detection of four (TevPV, YepPV, GroPV, YBPV) viruses was negatively associated with the relative abundance of grey-headed flying foxes (Table 2). Two of these viruses (TevPV and GroPV) were also positively associated with the relative abundance of black flying foxes. The negative associations between viral detections and either rainfall in the preceding month and/or water vapour pressure for all viruses except HerPV may reflect more frequent detections of these viruses during dry winter months. We identified significant negative associations between average NDVI and detection of YepPV and GroPV. Shedding of TevPV, GroPV, HeV and YBPV was significantly higher in 2011 compared with 2010. No effect of species was observed when flying fox species presence/absence data were used (Tables S3–S4).

**Table 2.** Results from stepwise regression to quantify associations between virus occurrence probabilities and biotic covariates (BFF (*Pteropus alecto*, Black flying fox), GHFF (*P. poliocephalus*, Grey-headed flying fox) and LRFF (Little red flying fox, *P. scapulatus*)) and abiotic covariates (mean Normalized Difference Vegetation Index (NDVI) within 20 km over the preceding 3 months, average precipitation in the preceding month, and average water vapour pressure, and year). Virus abbreviations as per Table 1.

	TevPV	YepPV	GroPV	HeV	MenV	HerPV^†^	YBPV
Intercept	−7.38 ***	−13.25 ***	−21.23	−6.92 ***	−20.07	−57.7	−5.73 ***
Avg precipitation (1mo)	−2.72 ***	−4.55 **	–	−3.32 *	0.97 *	−19.78	−0.95
Vapour pressure	–	–	−0.6 *	–	−1.45 **	−3.22	−0.74 *
Avg NDVI (3mo)	–	−2.26 *	−1.47 *	–	–	–	–
BFF	0.94 ***	1.64	1.19 *	–	–	–	–
GHFF	−1.59 ***	−6.61 ***	−2.69 **	–	−1.05	−24.55	−1.22 **
LRFF	–	–	−115.85	–	−114.68	–	–
Year 2011	2.71 ***	–	1.58 *	2.35 **	–	–	0.9 *
Year 2012	–	–	–	–	–	11.78	–
Location VIC	–	–	−20.4	−18.85	−20.86	–	1.12

Table shows final terms after backward stepwise selection, along with their coefficients and significance (*p < 0.05; **p < 0.001; ***p < 0.0001). – represents that the term was not included in the final model. Note that coefficients are from a binary logistic regression and can be interpreted as effects on a virus’ log odds of detection. For year and location, 2010 and Queensland represent the reference category, respectively. Colour scale ranges from dark blue (strong positive effect size) to dark red (strong negative effect size). **^†^**Poor model fit.

## Discussion

High-prevalence shedding of HeV from flying foxes [19] and unprecedented numbers of HeV spillovers to horses [17] were observed in the Austral winter of 2011. Alongside this, yet undetected at the time, we show that there was a synchronous shedding “pulse” of multiple bat paramyxoviruses.

We show that, while mean viral prevalence is low, multi-viral shedding from flying fox populations is common. Multi-viral shedding infrequently coalesces into an extreme, brief and spatially restricted shedding pulse, with potential consequences for spillover of multiple pathogens to humans and domestic animal hosts. In addition to HeV, one other virus detected here (MenPV) is known to be zoonotic [32]. However, the zoonotic potential of most of the other viruses is yet to be characterized. Beyond this, the distribution, dynamics and spillover potential of the broader viral community in Australian flying foxes is otherwise largely unknown. Improved understanding will arise via broadening the scope of spatiotemporal bat viral studies beyond single viruses and through further investigation of the large number of undiagnosed encephalitic deaths in domestic animals, for example, horses showing clinical signs consistent with HeV but that test HeV-negative [50].

The infrequent and spatially restricted nature of these intense pulses indicates they occur in response to complex ecological or epidemiological drivers, potentially over multi-year time scales. Longitudinal surveillance with short sampling intervals is required to fully elucidate these drivers, as studies undertaken for 1-2 years at a time (matching grant and student funding cycles) risk missing these intense pulses, and thereby underestimating their importance in driving spillover. Similarly, short-duration pulses may go unobserved or underestimated with infrequent sampling intervals, or during spillover-response sampling of wildlife hosts, which often begins months after the original spillover into human or animal populations has occurred [51]. More data are required to statistically derive ideal sampling frequency and duration, however, given the observed patterns here and a 2-7 year duration of climatic cycles [52], sampling at least every 1-2 months over at least 2 years would be required to more confidently elucidate drivers of transmission.

Previous studies have reported detection of multiple viruses in individual or pooled samples from bats [27,52–54]. These co-detections are often reported as incidental findings. We demonstrate that investigation of the spatiotemporal patterns of co-occurrence of these viruses within populations (co-circulation) and within hosts (co-infection) can likely provide insight into unobserved interactions between viruses and their hosts, and among virus pairs. Understanding these interactions requires an ecological framework. For example, viral diversity and abundance within and among hosts are expected to be governed by the fundamental processes of ecological drift, dispersal, selection, and speciation [8,55]. Drift drives increased diversity (though more so in hosts with limited dispersal); dispersal can drive increased transmission, coexistence and co-infection rates [8] and selection can drive viral community structure, particularly during periods of resource limitation.

Conditions at particular roosts at a given time may provide insight into broader drivers. For example, the detection of high-prevalence shedding of eight unique viruses from the Boonah roost in 2011 within weeks of an associated HeV spillover to a nearby horse suggests localized environmental conditions forcing multi-viral transmission or perhaps driving immune compromise and shedding from persistently infected individuals [26]. While 1–2 HeV spillovers had been detected annually since 2006, this case near Boonah was one of 18 spillovers detected in an unprecedented 12-week period in the winter of 2011 (Figure S8). This event, and a localized peak of four spillovers (including three in four weeks) in southeast Queensland and northeast New South Wales in 2017, are hypothesized to be linked to habitat loss and climatic events, where a steep rise in the southern oscillation index results in an acute food shortage for flying foxes [56]. The processes linking flying fox food shortages and HeV spillovers could also drive multi-viral spillover. However, more work is needed to define the mechanisms.

Most viral-viral interactions were positive, suggesting a greater frequency of facilitative than competitive interactions. Similarly, we speculate here that comparing the magnitudes of interaction coefficients across sampling levels provides further insight into the interactions within virus pairs – though future studies could use simulation models to explore these interaction types theoretically. For example, two endemic viruses that are never co-detected within an individual as a result of competitive interactions and/or cross-immunity would be expected to show a strong negative interaction coefficient at the *sample* level (as seen with HeV and YepPV). The theoretical expectation is for these viruses to show competitive, out-of-sync dynamics [11,57], however, how this manifests as a session level correlation may depend on prevalence and the relative influence of broader environmental drivers of transmission. Conversely, the frequent co-detection of a pair of viruses within a single individual (as a result of facilitation and cross-enhancement of infection) would result in a strong positive coefficient at the *sample* level (as observed between TevPV and HeV/YepPV). In this situation, synchronized dynamics with other viruses would be expected at a *session* level, though the strength of the interaction coefficient may also depend on viral prevalence (11). The frequent sample level co-detection of TevPV with all other viruses (highest median interaction coefficients) suggests it may play a central role in driving co-infections, potentially through immunosuppression or perhaps through activation of latent infections, as has been observed in herpesvirus co-infections [58]. Alternatively, a recent study has demonstrated that interactions between glycoproteins in HeV-NiV co-infections may promote viral entry and, perhaps, increase viral loads [59]. Further empirical and modelling studies are warranted to assess whether this approach could contribute to improved spillover predictions.

While data presented here suggest facilitative and competitive interactions among viruses, the interpretations are speculative. Larger sample sizes or simulation studies are required to test these predictions rigorously. In particular, samples collected from individual bats in the wild, along with experimental infection studies, are required to specifically investigate interactions among multiple pathogens. Additionally, here we consider only known paramyxoviruses. Unknown paramyxoviruses and other viral families likely also contribute to viral community interactions – future metagenomic studies to identify the broader viral community would be valuable, though specific viral PCRs and serological studies will be required to study the population dynamics and interactions of each virus over space and time. Here, samples were from urine collected from plastic sheets placed under bat roosts, making it likely that multiple bats, often of more than one species, contributed to each sample [60]. Similarly, bat roost counts used in this study are approximates only; detailed roost counts and understanding of species, age and sex roosting structures are required to fully understand the population-level drivers of co-circulation. Moreover, whilst the multiplexed fluid-based assay used here has comparable sensitivity to RT-qPCR assays on control samples [37], the reduced sensitivity of HeV in field samples, where multiple viruses may be present and competing for reagents within the one sample warrants further investigation with specific assays and incorporation into models. Regardless, the reduced assay sensitivity indicates that detections here likely represent viruses present in high viral titres, and therefore that the shedding pulse is an underestimate of the total viral load. Finally, more detailed data would be necessary to better disentangle biotic interactions among viruses from coincidental responses to variable environmental conditions, since both of these drivers can result in very similar co-occurrence patterns [45].

Our understanding of the known and unknown community of bat viruses (including in families other than paramyxoviruses) and their interactions is currently limited. Here, we show that multiple paramyxoviruses, including HeV, are synchronously shed in intense pulses from bats in Australia, but more in-depth study is necessary to quantify the exact nature and direction of interactions between specific pairs of viruses. These results are likely to be relevant globally and suggest that understanding the underlying and interacting mechanisms driving total viral load in bat populations is critical to predict and manage emerging viruses in bats and facilitate more rapid and cost-effective responses in the event of their spillover. Integrated, multidisciplinary studies, incorporating the ecology of the host and its entire community of pathogens, are essential to tackle these complex biological interactions and fully understand the dynamics of these emerging infections.

## Supplementary Material

Supplemental Material

## References

[1] Viney ME, Graham AL. Patterns and processes in parasite co-infection. Adv Parasitol. 2013;82:321–369. doi: 10.1016/B978-0-12-407706-5.00005-8 23548088

[2] Rohani P, Green CJ, Mantilla-Beniers NB, et al. Ecological interference between fatal diseases. Nature. 2003;422:885–888. doi: 10.1038/nature01542 12712203

[3] Abu-Raddad LJ, Patnaik P, Kublin JG. Dual infection with HIV and malaria fuels the spread of both diseases in sub-saharan Africa. Science. 2006;314:1603–1606. doi: 10.1126/science.1132338 17158329

[4] Lello J, Boag B, Fenton A, et al. Competition and mutualism among the gut helminths of a mammalian host. Nature. 2004;428:840–844. doi: 10.1038/nature02490 15103373

[5] Lloyd-Smith JO, Schreiber SJ, Kopp PE, et al. Superspreading and the effect of individual variation on disease emergence. Nature. 2005;438:355–359. doi: 10.1038/nature04153 16292310 PMC7094981

[6] Wells K, Fordham DA, Brook BW, et al. Disentangling synergistic disease dynamics: implications for the viral biocontrol of rabbits. J Anim Ecol. 2018;87:1418–1428. doi: 10.1111/1365-2656.12871 30133819

[7] Gorsich EE, Etienne RS, Medlock J, et al. Opposite outcomes of coinfection at individual and population scales. PNAS. 2018;115:7545–7550. doi: 10.1073/pnas.1801095115 29967175 PMC6055155

[8] Seabloom EW, Borer ET, Gross K, et al. The community ecology of pathogens: coinfection, coexistence and community composition. Ecol Lett. 2015;18:401–415. doi: 10.1111/ele.12418 25728488

[9] Graham AL. Ecological rules governing helminth-microparasite coinfection. PNAS. 2008;105:566–570. doi: 10.1073/pnas.0707221105 18182496 PMC2206576

[10] Kishida N, Sakoda Y, Eto M, et al. *Staphylococcus aureus* or *Haemophilus paragallinarum* exacerbates H9N2 influenza a virus infection in chickens. Arch Virol. 2004;149:2095–2104. doi: 10.1007/s00705-004-0372-1 15503199

[11] Vasco DA, Wearing HJ, Rohani P. Tracking the dynamics of pathogen interactions: Modeling ecological and immune-mediated processes in a two-pathogen single-host system. J Theor Biol. 2007;245:9–25. doi: 10.1016/j.jtbi.2006.08.015 17078973

[12] Alter MJ. Epidemiology of viral hepatitis and HIV co-infection. J Hepatol. 2006;44:S6–S9. doi: 10.1016/j.jhep.2005.11.004 16352363

[13] Bhattacharyya S, Gesteland PH, Korgenski K, et al. Cross-immunity between strains explains the dynamical pattern of paramyxoviruses. PNAS. 2015;112:13396–13400. doi: 10.1073/pnas.1516698112 26460003 PMC4629340

[14] El ME Z, Chander Y, Redig PT, et al. Selective isolation of avian influenza virus (AIV) from cloacal samples containing AIV and newcastle disease virus. J Vet Diagn Invest. 2011;23:330–332. doi: 10.1177/104063871102300222 21398457

[15] Hayman DTS, Bowen RA, Cryan PM, et al. Ecology of zoonotic infectious diseases in bats: current knowledge and future directions. Zoonoses Public Hlth. 2013;60:2–21. doi: 10.1111/zph.12000 PMC3600532 22958281

[16] Glennon EE, Restif O, Sbarbaro SR, et al. Domesticated animals as hosts of henipaviruses and filoviruses: a systematic review. Vet J. 2018;233:25–34. doi: 10.1016/j.tvjl.2017.12.024 29486875

[17] Plowright RK, Eby P, Hudson PJ, et al. Ecological dynamics of emerging bat virus spillover. Proc Biol Sci. 2015;282:20142124–20142124. doi: 10.1098/rspb.2014.2124 25392474 PMC4262174

[18] Halpin K, Hyatt AD, Fogarty R, et al. Pteropid bats are confirmed as the reservoir hosts of henipaviruses: a comprehensive experimental study of virus transmission. Am J Trop Med Hyg. 2011;85:946–951. doi: 10.4269/ajtmh.2011.10-0567 22049055 PMC3205647

[19] Field HE, Jordan D, Edson D, et al. Spatiotemporal aspects of Hendra virus infection in pteropid bats (flying-foxes) in Eastern Australia. PLoS ONE. 2015;10:e0144055. doi: 10.1371/journal.pone.0144055 26625128 PMC4666458

[20] Edson D, Field HE, McMichael LA, et al. Routes of Hendra virus Excretion in naturally-infected flying-foxes: implications for viral transmission and spillover risk. PLoS ONE. 2015;10:e0140670. doi: 10.1371/journal.pone.0140670 26469523 PMC4607162

[21] Goldspink LK, Edson DW, Vidgen ME, et al. Natural Hendra virus infection in flying-foxes - tissue tropism and risk factors. PLoS ONE. 2015;10:e0128835. doi: 10.1371/journal.pone.0128835 26060997 PMC4465494

[22] Martin G, Plowright R, Chen C, et al. Hendra virus survival does not explain spillover patterns and implicates relatively direct transmission routes from flying foxes to horses. J Gen Virol. 2015;96:1229–1237. doi: 10.1099/vir.0.000073 25667321 PMC7346679

[23] Paez DJ, Giles J, McCallum H, et al. Conditions affecting the timing and magnitude of Hendra virus shedding across pteropodid bat populations in Australia. Epidemiol Infect. 2017;57:1–11. 10.1017/S0950268817002138 PMC5783192 28942750

[24] Martin G, Yáñez-Arenas C, Plowright RK, et al. Hendra virus spillover is a bimodal system driven by climatic factors. Ecohealth. 2018;9:1–17. 10.1007/s10393-017-1309-y 29349533

[25] Giles JR, Eby P, Parry H, et al. Environmental drivers of spatiotemporal foraging intensity in fruit bats and implications for Hendra virus ecology. Sci Rep 2018;8:9555. doi: 10.1038/s41598-018-27859-3 29934514 PMC6015053

[26] Plowright RK, Peel AJ, Streicker DG, et al. Transmission or within-host dynamics driving pulses of zoonotic viruses in reservoir-host populations. PLoS Negl Trop Dis. 2016;10:e0004796. doi: 10.1371/journal.pntd.0004796 27489944 PMC4973921

[27] Barr JA, Smith C, Smith I, et al. Isolation of multiple novel paramyxoviruses from pteropid bat urine. J Gen Virol. 2015;96:24–29. doi: 10.1099/vir.0.068106-0 25228492

[28] Marsh GA, de Jong C, Barr JA, et al. Cedar virus: a novel henipavirus Isolated from Australian bats. PLoS Pathog. 2012;8:e1002836. doi: 10.1371/journal.ppat.1002836 22879820 PMC3410871

[29] Burroughs AL, Tachedjian M, Crameri G, et al. Complete Genome sequence of Teviot paramyxovirus, a novel Rubulavirus Isolated from Fruit bats in Australia. Genome Announc. 2015;3:e00177–15. doi: 10.1128/genomeA.00177-15 25883275 PMC4400418

[30] Kohl C, Tachedjian M, Todd S, et al. Hervey virus: study on co-circulation with Henipaviruses in Pteropid bats within their distribution range from Australia to Africa. PLoS ONE. 2018;13:e0191933. doi: 10.1371/journal.pone.0191933 29390028 PMC5794109

[31] Johnson R, Tachedjian M, Rowe B, et al. Alston virus, a novel paramyxovirus Isolated from bats Causes Upper respiratory Tract infection in Experimentally Challenged Ferrets. Viruses. 2018;10:675. doi: 10.3390/v10120675 30487438 PMC6315912

[32] Philbey AW, Kirkland PD, Ross AD, et al. An apparently new virus (family Paramyxoviridae) infectious for pigs, humans, and fruit bats. Emerg Infect Dis. 1998;4:269–271. doi: 10.3201/eid0402.980214 9621197 PMC2640116

[33] Chua KB, Bellini WJ, Rota PA, et al. Nipah virus: a recently emergent deadly paramyxovirus. Science. 2000;288:1432–1435. doi: 10.1126/science.288.5470.1432 10827955

[34] Chua KB, Wang L-F, Lam SK, et al. Tioman virus, a novel paramyxovirus isolated from Fruit bats in Malaysia. Virology. 2001;283:215–229. doi: 10.1006/viro.2000.0882 11336547

[35] Reich NG, Shrestha S, King AA, et al. Interactions between serotypes of dengue highlight epidemiological impact of cross-immunity. J R Soc Interface. 2013;10:20130414. doi: 10.1098/rsif.2013.0414 23825116 PMC3730691

[36] Burroughs AL, Durr PA, Boyd V, et al. Hendra virus infection dynamics in the grey-headed flying fox (Pteropus poliocephalus) at the southern-most Extent of Its range: further Evidence this species does not readily transmit the virus to horses. PLoS ONE. 2016;11:e0155252. doi: 10.1371/journal.pone.0155252 27304985 PMC4909227

[37] Boyd V, Smith I, Crameri G, et al. Development of multiplexed bead arrays for the simultaneous detection of nucleic acid from multiple viruses in bat samples. J Virol Methods. 2015;223:5–12. doi: 10.1016/j.jviromet.2015.07.004 26190638 PMC7113788

[38] Harris DJ. Inferring species interactions from co-occurrence data with Markov networks. Ecol. 2016;97:3308–3314. doi: 10.1002/ecy.1605 27912022

[39] Clark NJ, Wells K, Lindberg O. Unravelling changing interspecific interactions across environmental gradients using Markov random fields. Ecol. 2018;99:1277–1283. doi: 10.1002/ecy.2221 29768661

[40] Kissling WD, Dormann CF, Groeneveld J, et al. Towards novel approaches to modelling biotic interactions in multispecies assemblages at large spatial extents. J Biogeogr. 2012;39:2163–2178. doi: 10.1111/j.1365-2699.2011.02663.x

[41] Clark NJ, Wells K, Dimitrov D, et al. Co-infections and environmental conditions drive the distributions of blood parasites in wild birds. J Anim Ecol. 2016;85:1461–1470. doi: 10.1111/1365-2656.12578 27561363

[42] Barner AK, Coblentz KE, Hacker SD, et al. Fundamental contradictions among observational and experimental estimates of non-trophic species interactions. Ecol. 2018;99:557–566. doi: 10.1002/ecy.2133 29385234

[43] Dayton PK. Competition, disturbance, and community organization: the provision and subsequent utilization of space in a rocky intertidal community. Ecol Monogr. 1971;41:351–389. doi: 10.2307/1948498

[44] Gravel D, Baiser B, Dune JA, et al. Bringing Elton and Grinnell together: a quantitative framework to represent the biogeography of ecological interaction networks. Ecography. 2019;42:401–415. doi: 10.1111/ecog.04006

[45] Dormann CF, Bobrowski M, Dehling DM, et al. Biotic interactions in species distribution modelling: 10 questions to guide interpretation and avoid false conclusions. Glob Ecol Biogeogr. 2018;483:205.

[46] Stone L, Roberts A. The checkerboard score and species distributions. Oecologia. 1990;85:74–79. doi: 10.1007/BF00317345 28310957

[47] Gotelli NJ. Null model analysis of species co-occurrence patterns. Ecol. 2000;81:2606–2621. doi: 10.1890/0012-9658(2000)081[2606:NMAOSC]2.0.CO;2

[48] Harris DJ. *Rosalia: exact inference for small binary markov networks. r package version 0.1. 0. Zenodo*. 2015.

[49] Giles JR, Plowright RK, Eby P, et al. Models of Eucalypt phenology predict bat population flux. Ecol Evol. 2016;6:7230–7245. doi: 10.1002/ece3.2382 27891217 PMC5115174

[50] Smith CS, McLaughlin A, Field HE, et al. Twenty years of Hendra virus: laboratory submission trends and risk factors for infection in horses. Epidemiol Infect. 2016;144:1–18. doi: 10.1017/S0950268815000722 27357144 PMC9150281

[51] Plowright RK, Becker DJ, McCallum H, et al. Sampling to elucidate the dynamics of infections in reservoir hosts. Philos Trans R Soc, B. 2019. doi:10.1098/rstb.2018.0336. PMC6711310 31401966

[52] Baker KS, Todd S, Marsh G, et al. Co-circulation of diverse paramyxoviruses in an urban African fruit bat population. J Gen Virol. 2012;93:850–856. doi: 10.1099/vir.0.039339-0 22205718 PMC3542712

[53] Mortlock M, Dietrich M, Weyer J, et al. Co-circulation and excretion dynamics of diverse rubula- and related viruses in Egyptian rousette bats from South Africa. Viruses. 2019;11:37. doi: 10.3390/v11010037 30626055 PMC6356502

[54] Anthony SJ, Epstein JH, Murray KA, et al. A strategy to estimate unknown viral diversity in mammals. mBio. 2013;4:e00598–13–e00598–13. doi: 10.1128/mBio.00598-13 24003179 PMC3760253

[55] Vellend M. Conceptual synthesis in community ecology. Q Rev Biol. 2010;85:183–206. doi: 10.1086/652373 20565040

[56] Peel AJ, Eby P, Kessler M, et al. Hendra virus spillover risk in horses: heightened vigilance and precautions being urged this winter. Aust Vet J. 2017;95:N20–N21.

[57] Gupta S, Swinton J, Anderson RM. Theoretical studies of the effects of heterogeneity in the parasite population on the transmission dynamics of malaria. Proc R Soc B. 1994;256:231–238. doi: 10.1098/rspb.1994.0075 7914705

[58] Vieira J, O'Hearn P, Kimball L, et al. Activation of Kaposi's Sarcoma-associated herpesvirus (human herpesvirus 8) lytic replication by human cytomegalovirus. J Virol. 2001;75:1378–1386. doi: 10.1128/JVI.75.3.1378-1386.2001 11152511 PMC114044

[59] Bradel-Tretheway BG, Zamora JLR, Stone JA, et al. Nipah and Hendra virus glycoproteins induce comparable homologous but distinct heterologous fusion phenotypes. J Virol. 2019;93:e00577–19. doi: 10.1128/JVI.00577-19 30971473 PMC6580972

[60] Giles JR, Peel AJ, Wells K, et al. Optimizing non-invasive sampling of an infectious bat virus. bioRxiv. 2018. DOI:10.1101/401968.

