# Supplementary material for: Synchronous shedding of multiple bat paramyxoviruses coincides with peak periods of Hendra virus spillover
Source: Emerg Microbes Infect. 2019 Sep 7;8(1):1314–23. doi: 10.1080/22221751.2019.1661217 (PMC6746281; doi:10.1080/22221751.2019.1661217)
Supplement: Supplemental Material [file TEMI_A_1661217_SM1080.docx]

**SUPPLEMENTARY INFORMATION:**

Synchronous shedding of multiple bat paramyxoviruses coincides with peak periods of Hendra virus spillover

Alison J. Peel, Konstans Wells, John Giles, Victoria Boyd, Amy Burroughs, Daniel Edson, Gary Crameri, Michelle Baker, Hume Field, Linfa Wang, Hamish McCallum, Raina Plowright, Nicholas Clark

**Supplementary Methods**

Table S1: Details of sampling sites and under-roost urine collection events from black flying foxes (BFF, *Pteropus alecto*), grey-headed flying foxes (GHFF, *P. poliocephalus*), spectacled flying foxes (SFF, *P. conspicillatus*) and little red flying foxes (LRFF, *P. scapulatus*) in Queensland (QLD) and Victoria (VIC). Dates are in the format (dd/mm/yyyy). * For Boonah samples, multi-viral PCR testing was performed on a subset of the samples from each session (up to 22 from each session).

| **State** | **Location** | **Date** | **Number of samples tested** | **Species** |
| --- | --- | --- | --- | --- |
| QLD | Boonah | 11/7/2011 | 20 (30)* | Mixed roost (BFF, GHFF) |
|  |  | 26/7/2011 | 20 (30)* | Single-species roost (BFF) |
|  |  | 10/8/2011 | 18 (27)* | Mixed roost (BFF, GHFF) |
|  |  | 20/12/2011 | 21 (29)* | Mixed roost (BFF, GHFF) |
|  |  | 12/1/2012 | 21 (23)* | Mixed roost (BFF, GHFF) |
|  |  | 2/2/2012 | 21 (30)* | Mixed roost (BFF, GHFF) |
|  |  | 9/3/2012 | 22 (30)* | Mixed roost (BFF, GHFF) |
|  |  | 13/4/2012 | 17 (30)* | Mixed roost (BFF, GHFF) |
|  |  | 4/5/2012 | 20 (30)* | Mixed roost (BFF, GHFF) |
|  |  | 17/5/2012 | 20 (30)* | Mixed roost (BFF, GHFF) |
|  |  | 15/6/2012 | 20 (30)* | Mixed roost (BFF, GHFF) |
|  |  | 4/7/2012 | 20 (30)* | Mixed roost (BFF, GHFF) |
|  |  | 8/8/2012 | 22 (30)* | Mixed roost (BFF, GHFF) |
|  |  | 11/9/2012 | 20 (30)* | Mixed roost (BFF, GHFF) |
|  |  | 10/10/2012 | 22 (30)* | Mixed roost (BFF, GHFF) |
|  |  | 13/11/2012 | 16 (30)* | Mixed roost (BFF, GHFF, LRFF) |
|  | Cedar Grove | 10/11/2010 | 10 | Single-species roost (LRFF) |
|  |  | 1/12/2010 | 10 | Mixed roost (BFF, GHFF) |
|  |  | 5/1/2011 | 11 | Mixed roost (BFF, GHFF) |
|  |  | 18/1/2011 | 10 | Mixed roost (BFF, LRFF) |
|  |  | 1/2/2011 | 10 | Mixed roost (BFF, GHFF) |
|  |  | 10/2/2011 | 10 | Mixed roost (BFF, GHFF) |
|  |  | 18/2/2011 | 10 | Mixed roost (BFF, GHFF) |
|  |  | 24/2/2011 | 9 | Mixed roost (BFF, GHFF) |
|  |  | 10/3/2011 | 10 | Mixed roost (BFF, GHFF) |
|  |  | 25/3/2011 | 10 | Mixed roost (BFF, GHFF) |
|  |  | 1/4/2011 | 10 | Single-species roost (BFF) |
|  |  | 8/4/2011 | 10 | Single-species roost (BFF) |
|  |  | 12/5/2011 | 9 | Single-species roost (BFF) |
| VIC | Geelong | 4/11/2010 | 10 | Single-species roost (GHFF) |
|  |  | 10/11/2010 | 10 | Single-species roost (GHFF) |
|  |  | 17/11/2010 | 10 | Single-species roost (GHFF) |
|  |  | 14/12/2010 | 10 | Single-species roost (GHFF) |
|  |  | 22/12/2010 | 10 | Single-species roost (GHFF) |
|  |  | 20/1/2011 | 10 | Single-species roost (GHFF) |
|  |  | 25/1/2011 | 10 | Single-species roost (GHFF) |
|  |  | 2/2/2011 | 10 | Single-species roost (GHFF) |
|  |  | 9/2/2011 | 10 | Single-species roost (GHFF) |
|  |  | 18/2/2011 | 10 | Single-species roost (GHFF) |
|  |  | 24/2/2011 | 10 | Single-species roost (GHFF) |
|  |  | 2/3/2011 | 10 | Single-species roost (GHFF) |
|  |  | 16/3/2011 | 10 | Single-species roost (GHFF) |
|  |  | 25/3/2011 | 9 | Single-species roost (GHFF) |
|  |  | 29/3/2011 | 10 | Single-species roost (GHFF) |
|  |  | 7/4/2011 | 10 | Single-species roost (GHFF) |
|  |  | 15/4/2011 | 10 | Single-species roost (GHFF) |
|  |  | 19/4/2011 | 10 | Single-species roost (GHFF) |
|  |  | 28/4/2011 | 10 | Single-species roost (GHFF) |
|  |  | 3/5/2011 | 10 | Single-species roost (GHFF) |
|  |  | 10/5/2011 | 10 | Single-species roost (GHFF) |
|  |  | 17/5/2011 | 10 | Single-species roost (GHFF) |
|  |  | 1/6/2011 | 10 | Single-species roost (GHFF) |
|  |  | 7/6/2011 | 10 | Single-species roost (GHFF) |
|  |  | 15/6/2011 | 10 | Single-species roost (GHFF) |
|  |  | 29/6/2011 | 10 | Single-species roost (GHFF) |
|  |  | 13/7/2011 | 10 | Single-species roost (GHFF) |
|  |  | 21/7/2011 | 10 | Single-species roost (GHFF) |
|  |  | 28/7/2011 | 10 | Single-species roost (GHFF) |
|  |  | 3/8/2011 | 10 | Single-species roost (GHFF) |
|  |  | 12/8/2011 | 10 | Single-species roost (GHFF) |
|  |  | 23/8/2011 | 10 | Single-species roost (GHFF) |
|  |  | 30/8/2011 | 10 | Single-species roost (GHFF) |
|  |  | 6/9/2011 | 10 | Single-species roost (GHFF) |
|  |  | 13/9/2011 | 10 | Single-species roost (GHFF) |
|  |  | 21/9/2011 | 10 | Single-species roost (GHFF) |
|  |  | 27/9/2011 | 10 | Single-species roost (GHFF) |
|  |  | 4/10/2011 | 10 | Single-species roost (GHFF) |
|  |  | 14/10/2011 | 10 | Single-species roost (GHFF) |
|  |  | 18/10/2011 | 10 | Single-species roost (GHFF) |
|  |  | 26/10/2011 | 10 | Single-species roost (GHFF) |
|  |  | 1/11/2011 | 10 | Single-species roost (GHFF) |
|  |  | 11/11/2011 | 10 | Single-species roost (GHFF) |
|  |  | 15/11/2011 | 10 | Single-species roost (GHFF) |
|  |  | 21/11/2011 | 10 | Single-species roost (GHFF) |
|  |  | 2/12/2011 | 10 | Single-species roost (GHFF) |
|  |  | 14/12/2011 | 10 | Single-species roost (GHFF) |
|  |  | 22/12/2011 | 10 | Single-species roost (GHFF) |
|  |  | 6/1/2012 | 10 | Single-species roost (GHFF) |
|  |  | 12/1/2012 | 10 | Single-species roost (GHFF) |
|  |  | 20/1/2012 | 10 | Single-species roost (GHFF) |
|  |  | 24/1/2012 | 10 | Single-species roost (GHFF) |
|  |  | 3/2/2012 | 10 | Single-species roost (GHFF) |
|  |  | 10/2/2012 | 10 | Single-species roost (GHFF) |
|  |  | 23/2/2012 | 10 | Single-species roost (GHFF) |
|  |  | 29/2/2012 | 10 | Single-species roost (GHFF) |
|  |  | 9/3/2012 | 10 | Single-species roost (GHFF) |
|  |  | 21/3/2012 | 5 | Single-species roost (GHFF) |
|  |  | 29/3/2012 | 10 | Single-species roost (GHFF) |
|  |  | 5/4/2012 | 10 | Single-species roost (GHFF) |
|  |  | 18/4/2012 | 10 | Single-species roost (GHFF) |
|  |  | 9/5/2012 | 10 | Single-species roost (GHFF) |
|  |  | 17/5/2012 | 10 | Single-species roost (GHFF) |
|  |  | 22/5/2012 | 8 | Single-species roost (GHFF) |
|  |  | 30/5/2012 | 10 | Single-species roost (GHFF) |
|  |  | 13/6/2012 | 10 | Single-species roost (GHFF) |
|  |  | 20/6/2012 | 10 | Single-species roost (GHFF) |
|  |  | 28/6/2012 | 10 | Single-species roost (GHFF) |
|  |  | 6/7/2012 | 10 | Single-species roost (GHFF) |

Sample analyses

One-step reverse transcription PCR (RT-PCR) was performed with Platinum Taq kit (Invitrogen, Carlsbad, USA) using family-specific primers. Unincorporated dNTPs and primers were removed by ExoSAP-IT treatment (Affymetrix, Santa Clara, USA). A target-specific primer extension (TSPE) PCR followed; this reaction initiated PCR extension of targeted regions with biotinylated dCTP nucleotides. The 5’ ends of the TSPE primers were designed to contain 24 base TAG sequences complementary to the unique bead sets and the rest of the primer sequence was designed to bind to targets within the PCR product. The incorporation of biotin labelled cytosine into the PCR products allows binding and detection by streptavidin-R-phycoerythrin (SA-PE). PCR products then underwent hybridisation to uniquely coded MagPlex-Tag bead sets. A conjugation step with SA-PE then followed and the Hybridised microspheres were analysed on the Bio-Plex Array system integrated with Bio-Plex Manager software (v 6.0) (Bio-Rad Laboratories, Inc., CA, USA), set at the high reporter channel (RP1). Fluorescent signals were expressed as the Median Fluorescence Intensity (MFI) from 100 beads of each target set per well.

Estimating viral co-occurrence probabilities

The framework to derive MRF networks of co-occurrence probabilities using presence/absence data was originally described by Ising (1) and was recently brought to the attention of community ecologists by Harris (2) in parallel with hierarchical multi-species co-occurrence models (3,4). Briefly, the probability of observing a presence/absence outcome vector $y$ for species in the community is estimated with the joint probability function

$${\begin{aligned} p\left( y ;\alpha, \beta\right) \propto\frac{1}{\psi(\Theta)}exp\left( \mu\right) \\ \mu=\sum_{i\in V} {\alpha_{i}y}_{i}+\sum_{(i,j)\in E} \beta_{ij}{y_{i}y}_{j} (1) \end{aligned}}$$

The summation of the $\alpha$ terms captures the contributions of each species’ presences to the joint probability (e.g. the vertices of the network), while the second summation represents contributions of conditional interactions between each pair of species *i* and *j* (e.g. the edges of the network). Specifically, $\beta$ terms capture co-occurrence probabilities, which represent pairwise conditional dependencies while accounting for all other interactions in the graph. Maximizing the joint likelihood can be cumbersome and computationally challenging, as this requires estimating a normalizing constant ($\psi(\Theta)$) that grows exponentially with the number of species (1,5).

Table S2: Primers used in this study. Reproduced with permission from Boyd et al 2015 (6)

| **Primer name** | **Sequence 5' to 3'** | **TSPE targets sequence 5' to 3' with anti-tags (bold)** | **Virus specificity** | **Virus abbreviation** |
| --- | --- | --- | --- | --- |
| PAR-F1 L-gene | GAAGGITATTGTCAIAARNTNTGGAC^a^ | **aactttctctctctattcttattt**-caaccatggttccgcgcagtcta | Teviot | TevPV |
| PAR-R L-gene | *^b^*GCTGAAGTTACIGGITCICCDATRTTNC^a^ | **tacacaatattcatcataactaac**-caggtctctggaccataagctc | Yeppoon | YepPV |
|  |  | **tctctttaaacacattcaacaata**-agttccaagatcattgagccaccagt | Grove | GroPV |
|  |  | **caatttacatttcactttcttatc**-caaggttacggcacaacatgaa | Cedar | CedV |
|  |  | **ttaacaacttatacaaacacaaac**-ttatattttgacaggctcagaatgaatc | Hendra | HeV |
|  |  | **acatcaaattctttcaatatcttc**-ccattgcgtaccaaaatagtatgga | Menangle | MenV |
|  |  | **cataatcaatttcaactttctact**-attctctctgcaaccgaggcaaactc | Hervey | HerPV |
|  |  | **tacttctttactacaatttacaac**-tgcttgtagcaatatatctactaccctg | Yarra Bend | YBPV |
|  |  | **ctatcatttatctctttctcaatt**-ctcaatcgtcctgttcaaatctgg | Geelong | GeePV |
|  |  | **taacttacacttaactatcatctt**-tgtcttatcagctactgaagcgggc | Tioman | TiV |
|  |  | **tactacttctataactcacttaaa**-gctttactttgaaaggttaaggatgaact | Nipah | NiV |

**Results**

**Figure S1** .Number of detections of each of nine bat paramyxoviruses at each site. Viruses from left to right: Hendra virus, Cedar virus, Yarra Bend paramyxovirus, Geelong paramyxovirus, Hervey paramyxovirus, Menangle virus, Yeppoon paramyxovirus, Grove paramyxovirus, Teviot paramyxovirus.

**Figure S2**. Sensitivity of Hendra virus detection in a Taqman RT-PCR assay (output = Ct value on the y-axis) vs. bead-based multiplex assay (output = lnMFI on the x-axis). In the RT-PCR assay, samples with a Ct value ≤40 were considered positive. Remaining samples were considered negative, but were not assigned a Ct value so have been plotted at Ct = 42 for visualisation. Results are coloured by their Taqman result (N = negative, P = positive). Grey dotted lines represent the cutoff for positivity for each assay (Taqman RT-PCR: Ct ≤ 40; bead-based multiplex assay: lnMFI < 5.99). Blue line shows a linear regression Ct value vs. lnMFI for samples positive on the RT-PCR assay (F(1, 60) = 45.78, P <0.001, R^2^ 0.42)

**Figure S3**. Estimated infection prevalence (with 95% confidence intervals) of each viral species at the sample and session level from intercept-only logistic regressions, demonstrating just how sparse the occurrence data is. Viruses: Yarra Bend paramyxovirus (YBPV), Hervey paramyxovirus (HerPV), Menangle virus (MenV), Hendra virus (HeV), Grove paramyxovirus (GroV), Yeppoon paramyxovirus (YepPV), and Teviot paramyxovirus (TevPV)

Figure S4: Proportion of RNA-positive pooled samples nine paramyxoviruses (colours) from two flying fox roosts in Queensland (QLD) and one in Victoria (VIC) from November 2010 until November, 2012. Grey 95% confidence intervals show months when sampling was conducted but there were no detections. Blue shading represents winter months. In Queensland, dates prior to June 2011 represents sampling from Cedar Grove, and after this date, sampling continued from the nearby, newly formed Boonah roost

**Figure S5:** Monthly summary of the number of viral detections per urine sample for nine paramyxoviruses (colours) from two flying fox roosts in Queensland (QLD) and one in Victoria (VIC). * = sampling performed but no detections, blanks = no sampling performed. Grey numbers represent sample sizes. QLD sites contained a mix of black flying foxes (*P. alecto*) and grey-headed flying foxes (*P. poliocephalus*), whereas VIC contained only *P. poliocephalus.*

**Figure S6:** Median, first and third quartiles and 95% confidence intervals of viral interaction coefficients at the sample (red) and session (blue) level.


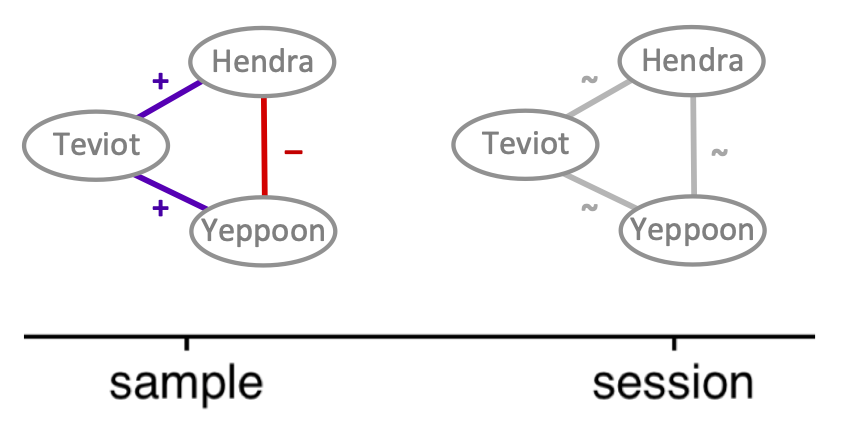


**Figure S7:** Schematic of positive (blue), negative (red) and neutral (grey) interactions between Hendra virus, Teviot paramyxovirus and Yeppoon paramyxovirus based on interaction Markov Random Field model coefficients.

**Table S3.** Results from stepwise regression to quantify associations between virus occurrence probabilities and biotic and abiotic covariates, with flying fox species presence/absence used instead of abundance. BFF (Pteropus alecto, Black flying fox), GHFF (P. poliocephalus, Grey-headed flying fox) and LRFF (Little red flying fox, P. scapulatus), mean Normalized Difference Vegetation Index (NDVI) within 20km over the preceding 3 months, average precipitation in the preceding month, and average water vapour pressure, and year. Table shows final terms after backward stepwise selection, along with their coefficients and significance (* p<0.05; ** p<0.001; *** p<0.0001). – represents that the term was not included in the final model. Note that coefficients are from a binary logistic regression and can be interpreted as effects on a virus’ log odds. For year and location, 2010 and Queensland represent the reference category, respectively. Colour scale ranges from dark blue (strong positive effect size) to dark red (strong negative effect size). †Poor model fit.

|  | **TevPV** | **YepPV** | **GroPV** | **HeV**† | **MenV**† | **HerV**† | **YBPV** |
| --- | --- | --- | --- | --- | --- | --- | --- |
| **Intercept** | -8.41 *** | -11.28 *** | -1.95 * | -23.55 | -22.97 | -39.56 | -3.84 *** |
| **Avg precipitation (1mo)** | -2.41 *** | – | – | -4.21 * | 0.88 * | – | -0.67 |
| **Vapour pressure** | – | -2.16 * | -0.96 *** | -1.44 * | -1.61 ** | -12.65 * | -1.2 *** |
| **Avg NDVI (3mo)** | – | -4.51 ** | -1.01 | – | – | -9.19 ** | – |
| **BFF** | – | – | – | – | – | – | – |
| **GHFF** | – | – | – | – | – | – | – |
| **LRFF** | – | – | – | – | – | – | – |
| **Year 2011** | 3.01 *** | 1.46 | 1.41 ** | – | 0.92 | – | 0.66 |
| **Year 2012** | – | – | – | – | – | – | – |
| **Location VIC** – | | – | -20.66 | – | – | – | – |

**Table S4** Full model results for Generalised linear regression models

### Using flying fox abundance counts

#### TevPV

## TevPV ~ avgprec + BFF + GHFF + year2011

| term | estimate | std.error | statistic | p.value |  |  |
| --- | --- | --- | --- | --- | --- | --- |
| (Intercept) | -7.3772788 | 0.7070929 | -10.433253 | 0.0000000 |  |  |
| avgprec | -2.7219877 | 0.5517540 | -4.933336 | 0.0000008 |  |  |
| BFF | 0.9387334 | 0.2756640 | 3.405354 | 0.0006608 |  |  |
| GHFF | -1.5871671 | 0.2983943 | -5.319027 | 0.0000001 |  |  |
| year2011 | 2.7136304 | 0.6897513 | 3.934216 | 0.0000835 |  |  |
| null.deviance | df.null | logLik | AIC | BIC | deviance | df.residual |
| 365.5346 | 1130 | -101.8458 | 213.6917 | 238.846 | 203.6917 | 1126 |

#### YepPV

## YepPV ~ avgprec + avgndvi + BFF + GHFF

| term | estimate | std.error | statistic | p.value |  |  |
| --- | --- | --- | --- | --- | --- | --- |
| (Intercept) | -13.246548 | 2.7842047 | -4.757749 | 0.0000020 |  |  |
| avgprec | -4.546437 | 1.6424738 | -2.768042 | 0.0056394 |  |  |
| avgndvi | -2.255921 | 0.8955903 | -2.518920 | 0.0117715 |  |  |
| BFF | 1.636987 | 1.0775707 | 1.519146 | 0.1287258 |  |  |
| GHFF | -6.607365 | 1.8424633 | -3.586158 | 0.0003356 |  |  |
| null.deviance | df.null | logLik | AIC | BIC | deviance | df.residual |
| 159.4844 | 1130 | -41.81225 | 93.6245 | 118.7788 | 83.6245 | 1126 |

#### GroPV

## GroPV ~ vapor + avgndvi + BFF + GHFF + LRFF + year2011 + locationGeelong

| term | estimate | std.error | statistic | p.value |  |  |
| --- | --- | --- | --- | --- | --- | --- |
| (Intercept) | -21.2266374 | 1163.7593736 | -0.0182397 | 0.9854476 |  |  |
| vapor | -0.6017256 | 0.2640281 | -2.2790209 | 0.0226658 |  |  |
| avgndvi | -1.4738447 | 0.6410202 | -2.2992171 | 0.0214926 |  |  |
| BFF | 1.1929151 | 0.4858812 | 2.4551580 | 0.0140823 |  |  |
| GHFF | -2.6945378 | 0.9005927 | -2.9919606 | 0.0027719 |  |  |
| LRFF | -115.8523333 | 8569.8093777 | -0.0135187 | 0.9892140 |  |  |
| year2011 | 1.5772396 | 0.6462470 | 2.4406142 | 0.0146623 |  |  |
| locationGeelong | -20.3979888 | 2463.2049042 | -0.0082811 | 0.9933927 |  |  |
| null.deviance | df.null | logLik | AIC | BIC | deviance | df.residual |
| 291.2537 | 1130 | -76.4218 | 168.8436 | 209.0905 | 152.8436 | 1123 |

#### HeV

## HeV ~ avgprec + year2011 + locationGeelong

| term | estimate | std.error | statistic | p.value |  |  |
| --- | --- | --- | --- | --- | --- | --- |
| (Intercept) | -6.915376 | 1.3483339 | -5.1288303 | 0.0000003 |  |  |
| avgprec | -3.321245 | 1.4457318 | -2.2972761 | 0.0216030 |  |  |
| year2011 | 2.354138 | 0.7983829 | 2.9486331 | 0.0031918 |  |  |
| locationGeelong | -18.845013 | 1672.9405346 | -0.0112646 | 0.9910123 |  |  |
| null.deviance | df.null | logLik | AIC | BIC | deviance | df.residual |
| 150.7964 | 1130 | -42.3169 | 92.63379 | 112.7572 | 84.63379 | 1127 |

#### MenV

## MenV ~ avgprec + vapor + GHFF + LRFF + locationGeelong

| term | estimate | std.error | statistic | p.value |  |  |
| --- | --- | --- | --- | --- | --- | --- |
| (Intercept) | -20.0731109 | 1.816627e+03 | -0.0110497 | 0.9911838 |  |  |
| avgprec | 0.9714333 | 4.342590e-01 | 2.2369905 | 0.0252870 |  |  |
| vapor | -1.4487967 | 4.934658e-01 | -2.9359618 | 0.0033252 |  |  |
| GHFF | -1.0545990 | 6.579926e-01 | -1.6027521 | 0.1089894 |  |  |
| LRFF | -114.6759840 | 1.337746e+04 | -0.0085723 | 0.9931604 |  |  |
| locationGeelong | -20.8573862 | 4.530008e+03 | -0.0046043 | 0.9963263 |  |  |
| null.deviance | df.null | logLik | AIC | BIC | deviance | df.residual |
| 168.0372 | 1130 | -55.33253 | 122.6651 | 152.8502 | 110.6651 | 1125 |

#### HerV

## HerPV ~ avgprec + vapor + GHFF + year2012

| term | estimate | std.error | statistic | p.value |  |  |
| --- | --- | --- | --- | --- | --- | --- |
| (Intercept) | -57.699041 | 37.307246 | -1.546591 | 0.1219620 |  |  |
| avgprec | -19.776849 | 16.087880 | -1.229301 | 0.2189589 |  |  |
| vapor | -3.223287 | 2.415118 | -1.334629 | 0.1819977 |  |  |
| GHFF | -24.551979 | 14.594688 | -1.682254 | 0.0925195 |  |  |
| year2012 | 11.782245 | 10.552713 | 1.116513 | 0.2642025 |  |  |
| null.deviance | df.null | logLik | AIC | BIC | deviance | df.residual |
| 159.4844 | 1130 | -35.1314 | 80.2628 | 105.4171 | 70.2628 | 1126 |

#### YBPV

## YBPV ~ avgprec + vapor + GHFF + year2011 + locationGeelong

| term | estimate | std.error | statistic | p.value |  |  |
| --- | --- | --- | --- | --- | --- | --- |
| (Intercept) | -5.7283415 | 0.7510683 | -7.626925 | 0.0000000 |  |  |
| avgprec | -0.9462209 | 0.5477643 | -1.727423 | 0.0840916 |  |  |
| vapor | -0.7434053 | 0.3398006 | -2.187769 | 0.0286864 |  |  |
| GHFF | -1.2232018 | 0.3884094 | -3.149259 | 0.0016368 |  |  |
| year2011 | 0.8996894 | 0.4505193 | 1.997005 | 0.0458246 |  |  |
| locationGeelong | 1.1154159 | 0.6599874 | 1.690056 | 0.0910172 |  |  |
| null.deviance | df.null | logLik | AIC | BIC | deviance | df.residual |
| 332.5973 | 1130 | -135.484 | 282.9681 | 313.1532 | 270.9681 | 1125 |

### Using flying fox presence/absence

#### TevPV (presence/absence)

## TevPV ~ avgprec + BFF_pa + year2011

| term | estimate | std.error | statistic | p.value |  |  |
| --- | --- | --- | --- | --- | --- | --- |
| (Intercept) | -8.411276 | 0.7103871 | -11.840412 | 0e+00 |  |  |
| avgprec | -2.411230 | 0.4899837 | -4.921041 | 9e-07 |  |  |
| BFF_pa | 3.094993 | 0.5172496 | 5.983559 | 0e+00 |  |  |
| year2011 | 3.006206 | 0.5075750 | 5.922683 | 0e+00 |  |  |
| null.deviance | df.null | logLik | AIC | BIC | deviance | df.residual |
| 365.5346 | 1130 | -103.0946 | 214.1892 | 234.3127 | 206.1892 | 1127 |

#### YepPV (presence/absence)

## YepPV ~ vapor + avgndvi + BFF_pa + GHFF_pa + LRFF_pa + year2011

| term | estimate | std.error | statistic | p.value |  |  |
| --- | --- | --- | --- | --- | --- | --- |
| (Intercept) | -11.277452 | 2.1641886 | -5.2109376 | 0.0000002 |  |  |
| vapor | -2.164906 | 0.8642400 | -2.5049821 | 0.0122458 |  |  |
| avgndvi | -4.514952 | 1.4427387 | -3.1294314 | 0.0017514 |  |  |
| BFF_pa | 9.971917 | 2.8072633 | 3.5521842 | 0.0003820 |  |  |
| GHFF_pa | -2.293136 | 1.0406837 | -2.2034894 | 0.0275603 |  |  |
| LRFF_pa | -16.893803 | 1531.6403432 | -0.0110299 | 0.9911996 |  |  |
| year2011 | 1.455482 | 0.9244656 | 1.5744033 | 0.1153943 |  |  |
| null.deviance | df.null | logLik | AIC | BIC | deviance | df.residual |
| 159.4844 | 1130 | -44.11642 | 102.2328 | 137.4488 | 88.23283 | 1124 |

#### GroPV (presence/absence)

## GroPV ~ vapor + avgndvi + GHFF_pa + LRFF_pa + year2011 + locationGeelong

| term | estimate | std.error | statistic | p.value |  |  |
| --- | --- | --- | --- | --- | --- | --- |
| (Intercept) | -1.9476267 | 0.8383846 | -2.3230707 | 0.0201754 |  |  |
| vapor | -0.9597859 | 0.2356687 | -4.0726068 | 0.0000465 |  |  |
| avgndvi | -1.0057131 | 0.5287231 | -1.9021547 | 0.0571509 |  |  |
| GHFF_pa | -1.0295957 | 0.5501056 | -1.8716328 | 0.0612574 |  |  |
| LRFF_pa | -18.3200381 | 4584.6153576 | -0.0039960 | 0.9968117 |  |  |
| year2011 | 1.4078231 | 0.5126746 | 2.7460362 | 0.0060320 |  |  |
| locationGeelong | -20.6641943 | 1074.3338147 | -0.0192344 | 0.9846541 |  |  |
| null.deviance | df.null | logLik | AIC | BIC | deviance | df.residual |
| 291.2537 | 1130 | -81.49654 | 176.9931 | 212.2091 | 162.9931 | 1124 |

#### HeV (presence/absence)

## HeV ~ avgprec + vapor + BFF_pa + GHFF_pa

| term | estimate | std.error | statistic | p.value |  |  |
| --- | --- | --- | --- | --- | --- | --- |
| (Intercept) | -23.548025 | 1621.4203808 | -0.0145231 | 0.9884127 |  |  |
| avgprec | -4.213683 | 1.7897924 | -2.3542858 | 0.0185583 |  |  |
| vapor | -1.443338 | 0.6215163 | -2.3222847 | 0.0202176 |  |  |
| BFF_pa | 17.156708 | 1621.4200145 | 0.0105813 | 0.9915575 |  |  |
| GHFF_pa | -1.434768 | 0.6105176 | -2.3500851 | 0.0187691 |  |  |
| null.deviance | df.null | logLik | AIC | BIC | deviance | df.residual |
| 150.7964 | 1130 | -41.13461 | 92.26922 | 117.4235 | 82.26922 | 1126 |

#### MenV (presence/absence)

## MenV ~ avgprec + vapor + BFF_pa + year2011

| term | estimate | std.error | statistic | p.value |  |  |
| --- | --- | --- | --- | --- | --- | --- |
| (Intercept) | -22.9728254 | 1057.3921317 | -0.0217259 | 0.9826666 |  |  |
| avgprec | 0.8771860 | 0.4253897 | 2.0620765 | 0.0392005 |  |  |
| vapor | -1.6140439 | 0.5104058 | -3.1622756 | 0.0015654 |  |  |
| BFF_pa | 18.8536573 | 1057.3920257 | 0.0178303 | 0.9857742 |  |  |
| year2011 | 0.9230311 | 0.5659832 | 1.6308455 | 0.1029229 |  |  |
| null.deviance | df.null | logLik | AIC | BIC | deviance | df.residual |
| 168.0372 | 1130 | -56.71007 | 123.4201 | 148.5744 | 113.4201 | 1126 |

#### HerV (presence/absence)

## HerPV ~ vapor + avgndvi + BFF_pa + GHFF_pa

| term | estimate | std.error | statistic | p.value |  |  |
| --- | --- | --- | --- | --- | --- | --- |
| (Intercept) | -39.563074 | 1865.605504 | -0.0212066 | 0.9830809 |  |  |
| vapor | -12.647572 | 6.386811 | -1.9802639 | 0.0476739 |  |  |
| avgndvi | -9.188089 | 3.221946 | -2.8517201 | 0.0043483 |  |  |
| BFF_pa | 33.737870 | 1865.592719 | 0.0180843 | 0.9855716 |  |  |
| GHFF_pa | -8.681081 | 3.535883 | -2.4551378 | 0.0140831 |  |  |
| null.deviance | df.null | logLik | AIC | BIC | deviance | df.residual |
| 159.4844 | 1130 | -34.47116 | 78.94233 | 104.0966 | 68.94233 | 1126 |

#### YBPV (presence/absence)

## YBPV ~ avgprec + vapor + GHFF_pa + year2011

| term | estimate | std.error | statistic | p.value |  |  |
| --- | --- | --- | --- | --- | --- | --- |
| (Intercept) | -3.8381096 | 0.6731047 | -5.702099 | 0.0000000 |  |  |
| avgprec | -0.6730755 | 0.4142291 | -1.624887 | 0.1041866 |  |  |
| vapor | -1.1982946 | 0.3248531 | -3.688727 | 0.0002254 |  |  |
| GHFF_pa | -0.8282614 | 0.5125001 | -1.616120 | 0.1060685 |  |  |
| year2011 | 0.6585778 | 0.4306729 | 1.529183 | 0.1262191 |  |  |
| null.deviance | df.null | logLik | AIC | BIC | deviance | df.residual |
| 332.5973 | 1130 | -140.949 | 291.898 | 317.0522 | 281.898 | 1126 |

**Figure S8:** Reported Hendra virus spillover events 1994 – 2019 (as of 1^st^ August, 2019). The division of events into the subtropics and tropics is based on the Tropic of Capricorn, with locations north of Rockhampton considered ‘Tropics’.


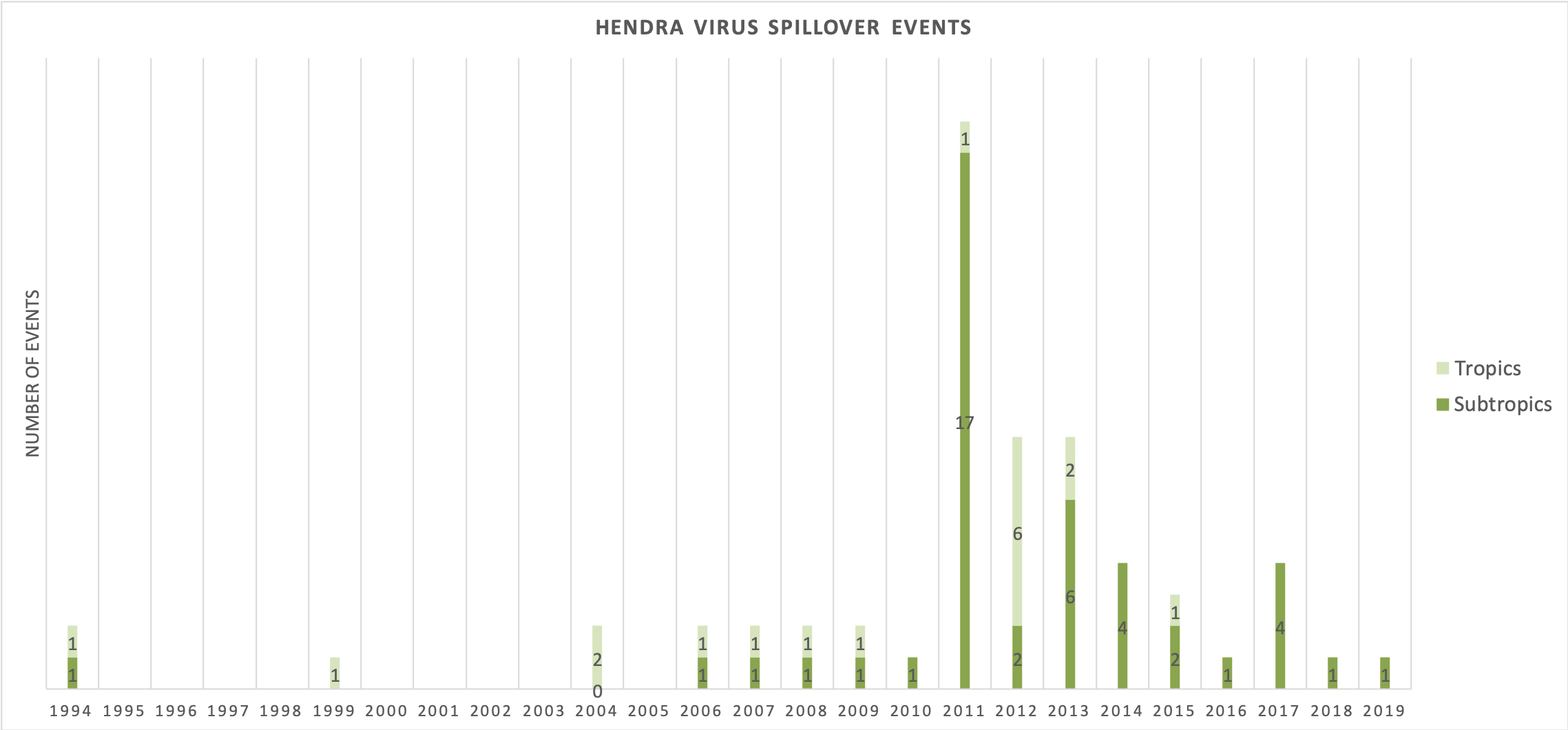


**References**

1. Ising E. Beitrag zur Theorie des Ferromagnetismus. Z Physik. Springer-Verlag; 1925 Feb;31(1):253–8.

2. Harris DJ. Inferring species interactions from co-occurrence data with Markov networks. Ecol. 2016 Dec 2;97(12):3308–14.

3. Ovaskainen O, Hottola J, Siitonen J. Modeling species co-occurrence by multivariate logistic regression generates new hypotheses on fungal interactions. Ecol. 2010 Sep;91(9):2514–21.

4. Warton DI, Blanchet FG, O'Hara RB, Ovaskainen O, Taskinen S, Walker SC, et al. So Many Variables: Joint Modeling in Community Ecology. Trends Ecol Evol. 2015 Dec;30(12):766–79.

5. Lindburg O. Markov Random Fields in Cancer Mutation Dependencies. Turku, Finland: fi=Turun yliopisto|en=University of Turku|; 2016 Jun 10.

6. Boyd V, Smith I, Crameri G, Burroughs AL, Durr PA, White J, et al. Development of multiplexed bead arrays for the simultaneous detection of nucleic acid from multiple viruses in bat samples. Journal of Virological Methods. 2015 Jul;223:5–12.
